# Comparison of image quality, arterial depiction, and radiation dose between two rapid kVp-switching dual-energy CT scanners in CT angiography at 40-keV

**DOI:** 10.1007/s11604-023-01448-5

**Published:** 2023-05-22

**Authors:** Tetsuro Kaga, Yoshifumi Noda, Shoma Nagata, Nobuyuki Kawai, Toshiharu Miyoshi, Fuminori Hyodo, Hiroki Kato, Masayuki Matsuo

**Affiliations:** 1 Department of Radiology, Gifu University, 1-1 Yanagido, Gifu, 501-1194 Japan; 2 Department of Radiology Services, Gifu University Hospital, Gifu, Japan; 3 Institute for Advanced Study, Gifu University, Gifu, Japan

**Keywords:** Dual-energy CT, Rapid kVp-switching, CT angiography, Noise power spectrum, Modulation transfer function

## Abstract

**Purpose:**

To compare the quantitative parameters and qualitative image quality of dual-energy CT angiography (CTA) between two rapid kVp-switching dual-energy CT scanners.

**Materials and methods:**

Between May 2021 and March 2022, 79 participants underwent whole-body CTA using either Discovery CT750 HD (Group A, *n* = 38) or Revolution CT Apex (Group B, *n* = 41). All data were reconstructed at 40-keV and with adaptive statistical iterative reconstruction-Veo of 40%. The two groups were compared in terms of CT numbers of the thoracic and abdominal aorta, and the iliac artery, background noise, signal-to-noise ratio (SNR) of the artery, CT dose-index volume (CTDI_vol_), and qualitative scores for image noise, sharpness, diagnostic acceptability, and arterial depictions.

**Results:**

The median CT number of the abdominal aorta (*p* = 0.04) and SNR of the thoracic aorta (*p* = 0.02) were higher in Group B than in Group A, while no difference was observed in the other CT numbers and SNRs of the artery (p = 0.09–0.23). The background noises at the thoracic (*p* = 0.11), abdominal (*p* = 0.85), and pelvic (*p* = 0.85) regions were comparable between the two groups. CTDI_vol_ was lower in Group B than in Group A (*p* = 0.006). All qualitative scores were higher in Group B than in Group A (*p* < 0.001–0.04). The arterial depictions were nearly identical in both two groups (*p* = 0.005–1.0).

**Conclusion:**

In dual-energy CTA at 40-keV, Revolution CT Apex improved qualitative image quality and reduced radiation dose.

## Introduction

Whole-body CT angiography (CTA) is widely used as a non-invasive imaging tool for understanding vascular anatomic features, diagnosing aortic diseases, specifying feeding arteries of the tumor or related arteries to active bleeding, and assessing before and after endovascular aortic aneurysm repair (EVAR) and transcatheter aortic valve implantation [1–7].

Virtual monochromatic images (VMIs) generated by dual-energy CT at 40-keV with reduced iodine dose can provide significantly higher CT attenuation than single-energy CT images at 120-kilovolt peak (kVp) [8]. Moreover, Shuman et al. [9] reported that VMIs at 50-keV reconstructed with an iterative reconstruction technique had comparable aortic CT attenuation, signal-to-noise ratio (SNR), and contrast-to-noise ratio to the single-energy CT images at 120-kVp while reducing 70% of iodine dose in abdominal dual-energy CTA. Generally, VMIs at low energy levels are annoying because they significantly increase image noise [10], however, evolving image reconstruction methods can provide lower image noise. Therefore, CTA imaging at 40-keV with reduced iodine dose has been established protocol [8, 11].

In 2010, GE Healthcare introduced the Gemstone Spectral Imaging (GSI) dual-energy CT platform, which was first mounted on Discovery CT750 HD. Their dual-energy CT scanners use a rapid kVp-switching system that can switch between tube voltages at 80- and 140-kVp. This platform, however, had several technical and clinical limitations such as incomplete energy separation between high- and low-energies and result in increasing image noise particularly on VMIs at low-keV and the relatively long reconstruction time. In recent years, the latest dual-energy CT scanner called Revolution CT Apex with the improved GSI platform (GSI-Xtream) has become available. This scanner improves image quality by reducing beam hardening artifacts and metal artifacts and increasing data collection accuracy through various hardware and firmware updates [12–14]. Beam hardening artifacts, also known as cupping artifacts, can reduce the accuracy of displayed CT numbers; however, the latest dual-energy CT scanners could provide more accurate CT numbers by more accurate correction of beam hardening artifacts [14]. We hypothesized that the latest dual-energy CT scanner could provide accurate CT numbers while also improving image quality. Thus, the purpose of this study was to compare the CT number, background noise, SNR, radiation dose, and qualitative image quality in dual-energy CTA between the latest and one generation earlier dual-energy CT scanners in 40-keV with reduced iodine dose setting.

## Materials and methods

### Phantom study

Before the clinical study, we performed two phantom studies. One was to verify the effect of beam hardening artifacts while another was to compare the objective image quality on VMIs at 40-keV between the two dual-energy CT scanners (Discovery CT750 HD and Revolution CT Apex; GE Healthcare, Milwaukee, WI). In each phantom study, we used the scan parameters described in the following “Dual-energy CT scanning protocol” section.

**Fig. 1:**
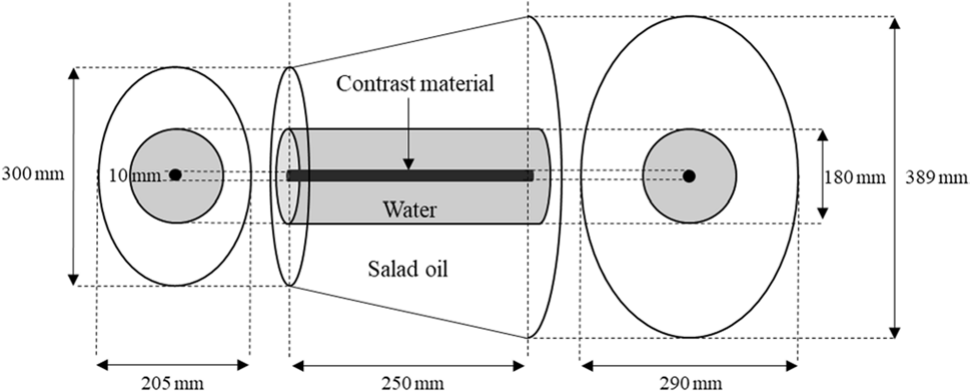
A schematic diagram of a self-made phantom

For the first phantom study, we used the self-produced elliptical cone phantom comprised a center-mounted acrylic tube with diluted contrast material which is surrounded by water and salad oil in a hermetic container (Fig. 1). Diluted contrast material was adjusted to 150 HU under conditions that single-energy CT scanning at 120-kVp was performed using Revolution CT Apex. The CT number was measured using a region-of-interest (ROI) placed at almost in the middle of the tube at intervals of 2.5 mm.

For the second phantom study, we used self-produced column phantom of 25 cm in diameter in which acrylic resin rod of 3 cm in diameter was centered and filled with water. Noise power spectrum (NPS) and modulation transfer function (MTF) were calculated using CTmeasure software (version 0.98f; Japanese Society of CT Technology, Hiroshima, Japan). NPS was calculated by the radial frequency method with a square ROI [15] on CT image of self-made water phantom. MTF was calculated by circular edge technique [16]. Moreover, signal difference to noise ratio (SDNR) was calculated by following equation [17]: SDNR = [(HU_water_ – HU_rod_)^2^ × MTF^2^ / NPS] ^1/2^, where HU_water_ denotes the mean CT values of water and HU_rod_ represents that of the acrylic resin rod.

### Participants

This prospective study was approved by the Institutional Review Board of our institution, and written informed consent was obtained from all participants prior to enrollment in this research. From May 2021 to March 2022, 84 consecutive participants underwent whole-body CTA using dual-energy CT scanners at our institution. Five participants were excluded from the study because of one of the following reasons: loss of CT raw data (*n* = 4) and subcutaneous leakage of contrast material during contrast injection (*n* = 1). Following the exclusions, the remaining 79 participants (55 men and 24 women, mean age, 71.1 ± 12.7 years; age range, 26–95 years; mean body weight, 60.8 ± 13.1 kg; body weight range, 34.0–97.5 kg; mean body mass index (BMI), 22.7 ± 4.2 kg/m^2^; BMI range, 14.0–37.8 kg/m^2^) were included in this study. These participants were scanned at random by either Discovery CT750 HD or Revolution CT Apex. Finally, 38 participants were scanned using the Discovery CT750 HD (Group A) and 41 were scanned using the Revolution CT Apex (Group B). Table 1 displays the clinical diagnoses.

**Table 1:** Background vascular diseases

Clinical diagnosis	Group A	Group B
Abdominal aortic aneurysm after EVAR	10	15
Thoracic aortic aneurysm after TEVAR	4	1
Abdominal aortic aneurysm after artificial blood vessel replacement surgery	8	7
After thoracic artificial blood vessel replacement surgery for thoracic aortic aneurysm, aortic dissection, or aortic valve-related disease	2	8
Aortic dissection	7	5
Thoracic and abdominal aortic aneurysms after TEVAR and EVAR	1	1
Thoracic aortic aneurysm after artificial blood vessel replacement surgery and abdominal aortic aneurysm after EVAR	0	2
Thoracic and abdominal aortic aneurysms after artificial blood vessel replacement surgery	1	0
Abdominal aortic aneurysm	1	0
Large vessel vasculitis	1	0
Normal study	2	0
Thoracic aortic aneurysm after TEVAR and abdominal aortic aneurysm after artificial blood vessel replacement surgery	1	0
Traumatic aortic rupture	0	1
Common iliac artery after artificial blood vessel replacement surgery	0	1
Total	38	41

*EVAR* Endovascular aortic aneurysm repair, *TEVAR* Thoracic endovascular aortic aneurysm repair

### Dual-energy CT scanning protocol

All examinations were carried out using a rapid kVp-switching dual-energy CT scanner, either Discovery CT750 HD or Revolution CT Apex. CT scans in the craniocaudal direction were obtained in the range of the supraclavicular fossa to the pubic symphysis. Table 2 summarizes the detailed technological features and scanning parameters. The Discovery CT750 HD had the following CT imaging parameters: detector row, 64; detector configuration, 64 detectors with 0.625 mm section thickness; beam width, 40 mm; rotation time, 0.6 s; helical pitch, 0.984:1; scan FOV, 320 mm; tube current, variable (GSI Assist; GE Healthcare, Milwaukee, WI); noise index, 10.0 HU at 5 mm slice collimation based on filtered back projection. Meanwhile, the following CT imaging parameters were used in the Revolution CT Apex: detector row, 256; detector configuration, 128 detectors with 0.625 mm section thickness; beam width, 80 mm; rotation time, 0.5 s; helical pitch, 0.508:1; scan FOV, 320 mm; tube current, variable (GSI Assist); noise index, 8.5 HU at 5 mm slice collimation based on adaptive statistical iterative reconstruction-Veo (ASiR-V) of 40%. Raw projection of arterial phase CT data at 1.25 mm section thickness with 50% overlap was reconstructed at 40-keV and with an ASiR-V of 40% in both groups.

**Table 2:** Technological differences of hardware, firmware, and acquisition parameters between Discovery CT750 HD and Revolution CT Apex, and contribution to image quality

Parameter	Discovery CT750 HD	Revolution CT Apex	Contribution to image quality
Detector row	64	256	N.A
Detector configuration	0.625-mm × 64	0.625-mm × 128	Decreasing motion artifact
Beam width (mm)	40	80	N.A
Rotation time (s)	0.6	0.5	N.A
Helical Pitch	0.984:1	0.508:1	Increasing spatial resolution
Scan FOV (mm)	320	320	N.A
Tube current (mA)	variable (GSI-Assist)	variable (Updated GSI-Assist)	Optimizing CTDI_vol_
Noise index (HU)	10.0 (based on FBP)	8.5 (based on ASiR-V of 40%)	N.A
GSI platform	GSI	GSI-Xtream	Decreasing image noise
X-ray tube	Performix™ HD	Quantix™ 160	Decreasing image noise
Scintillator Material	Gemstone	Gemstone	N.A
3D Collimator	N.A	Yes	Decreasing scattered X-rays
Data acquisition system	Volara HD digital DAS	Clarity DAS	Decreasing electronic noise
Image reconstruction kernel	Standard kernel	Standard kernel	N.A

*N.A.* not applicable, *FOV* field of view, *GSI* Gemstone spectral imaging, *CTDIvol* CT dose-index volume, *FBP* filtered back projection, *ASiR-V* adaptive statistical iterative reconstruction-Veo, *DAS* Digital Acquisition System

The contrast material (Iohexol 240 mgI/mL; GE Healthcare Pharma, Tokyo, Japan), which was diluted by 80% with simultaneous injection of saline, was intravenously injected at 4 mL/s. As a circular ROI, a 15–20 mm diameter circle was placed in the descending aorta at the level of the bronchial carina. Real-time fluoroscopic monitoring scans (140-kVp, 10 mA) were initiated 5 s after contrast injection. The contrast injection was discontinued when the bolus-tracking technique (SmartPrep; GE Healthcare) detected contrast enhancement reaching 80 HU and was followed by 20 mL saline chaser at a same rate, which was used in the previous study [18]. After the bolus-tracking program detected a threshold attenuation of 80 HU, CT scanning for the arterial phase was started with a 5 s scan delay.

The CT dose-index volume (CTDI_vol_) was recorded from the radiology information system.

### Quantitative image analysis

Using a commercially available Digital Imaging and Communications in Medicine viewer, a radiologist (T.K., with 4 years of post-training experience in interpreting CT images) measured the CT numbers of the thoracic aorta (mean CT numbers of the ascending aorta, aortic arch, and descending aorta), abdominal aorta (mean CT numbers of the upper, middle, and lower abdominal aorta), and iliac arteries (mean CT numbers of the bilateral common iliac arteries). In cases of after thoracic endovascular aortic aneurysm repair (TEVAR), CT number measurements in the part of stent placement were waived to avoid the influence of metal artifact. In cases of after EVAR, CT number measurements in the part of stent placement were waived, and those in the common iliac artery were alternately measured in the external iliac artery to avoid the influence of metal artifacts. In cases of communicating aortic dissection, CT number measurements were performed in true lumen. The radiologist placed a circular ROI of 5–30 mm in diameter on axial images, encompassing as much of the vascular lumen as possible while avoiding the vascular walls, calcification, thrombus, and artifacts. The background noise for each participant was one standard deviation of the CT numbers of homogeneous subcutaneous fat tissue at the level of the carina, upper pole of the left kidney, and cranial aspect of the femoral head representative of the thoracic, abdominal, and pelvic regions. The SNR was determined by dividing the CT number of each vessel by the background noise at corresponding anatomic regions.

### Qualitative image analysis

Two radiologists (T.K. and S.N., with 4 and 6 years of post-training experience in interpreting CT images, respectively), who were unaware of the CT scanners used, independently and randomly reviewed the axial images and graded the image quality in terms of subjective image noise, image sharpness, and diagnostic acceptability using a 5-point Likert scale (Table 3) [19]. Axial images were initially presented with a window setting preset at 350 HU of the window width and 40 HU of the window level; however, the reviewers were allowed to adjust the window setting at their discretion during evaluations.

**Table 3:** Grading scales for the qualitative image analyses

Grading score	Subjective image noise	Image sharpness	Diagnostic acceptability
1	Unacceptable noise	Blurry	Unacceptable
2	Above average	Worse than average	Poor
3	Average	Average	Average
4	Less than average	Better than average	High
5	Minimal	Sharpest	Excellent

The radiologists reviewed the axial images and graded the arterial depictions of the brachiocephalic, common carotid, subclavian, bronchial, internal thoracic, intercostal, common hepatic, proper hepatic, splenic, left gastric, gastroduodenal, inferior phrenic, superior mesenteric, inferior mesenteric, renal, lumbar, common iliac, external iliac, internal iliac, iliolumbar, superior gluteal, inferior gluteal, obturator, and inferior epigastric arteries using a 5-point rating scale as follows, which were used in the previous study [8]: 5, all vascular segments were visualized from the trunk to the subsegmental peripheral artery; 4, intermediate between Grades 5 and 3; 3, nearly half of all vascular segments were visualized; 2, intermediate between Grades 3 and 1; and 1, none of the vascular segments were visualized. When multiple blood vessels were applicable, the one with the best depiction was chosen.

### Statistical analysis

The MedCalc statistical software for Windows was used to conduct the statistical analyses (MedCalc software v.20.114; Mariakerke, Belgium). The two-sample *t* and Fisher’s tests were used to compare the differences in participants’ age, sex, height, body weight, and BMI between the two groups. The Mann–Whitney *U* test was used to compare the differences in terms of injected contrast volume, injected amount of iodine, CTDI_vol_, CT numbers of the arteries, background noise, SNR, and qualitative rating scores between the two groups. A *p*-value of less than 0.05 was considered to be statistically significant.

Inter-observer variability in qualitative analyses was assessed using the *ĸ* statistics. A *ĸ*-value of ≤ 0.20 was interpreted as slight agreement, 0.21–0.40 as fair agreement, 0.41–0.60 as moderate agreement, 0.61–0.80 as substantial agreement, and ≥ 0.81 as almost perfect agreement [20].

## Results

### Phantom study

**Fig. 2:**
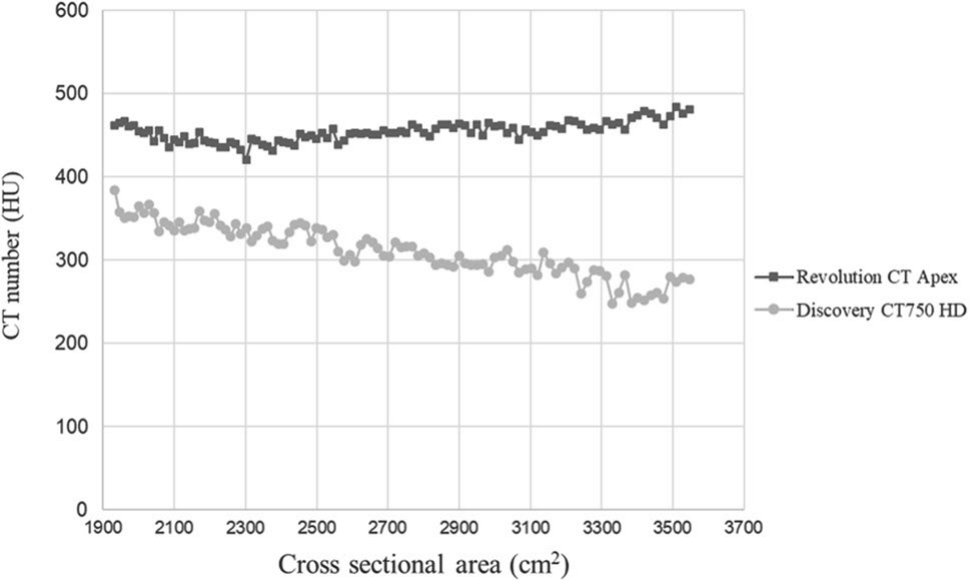
Graphs showing CT numbers of the phantom in the two dual-energy CT scanners

**Fig. 3:**
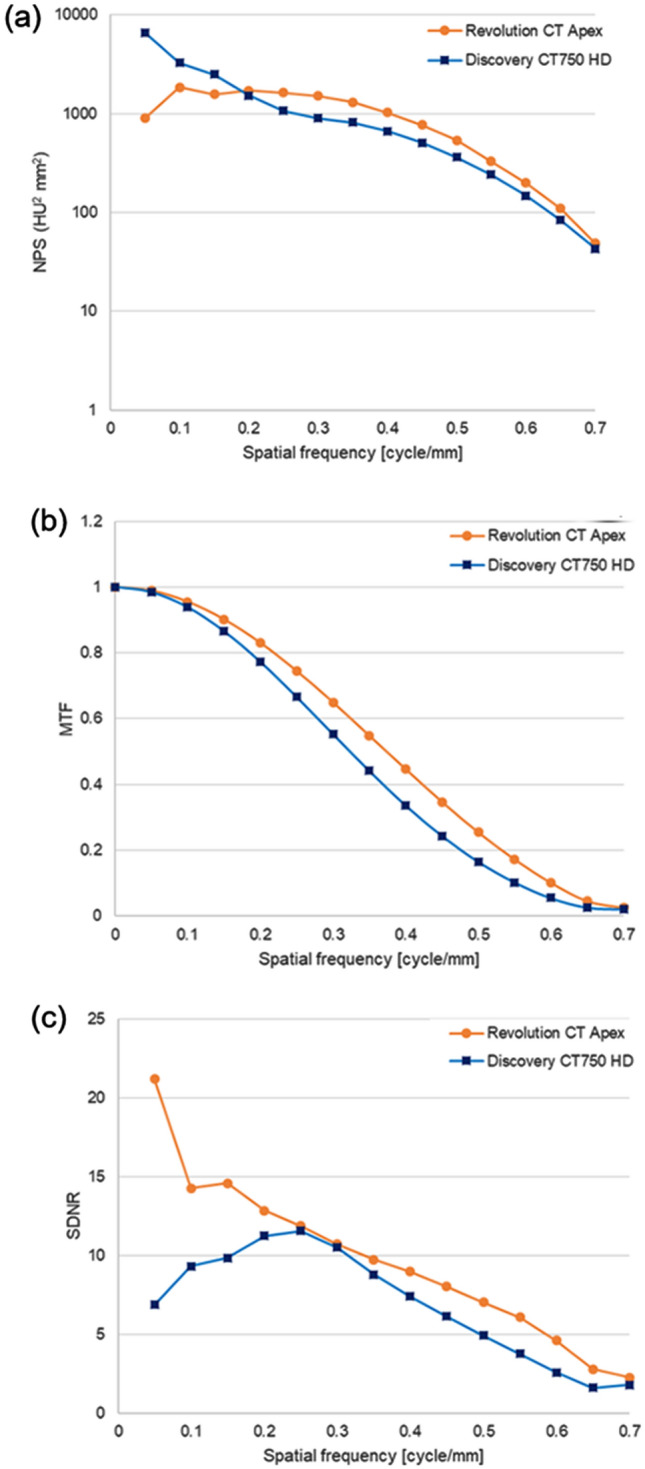
**a** Noise power spectrum (NPS), **b** modulation transfer function (MTF), and **c** signal difference to noise ratio (SDNR) curves of phantom study scanned using two dual-energy CT scanners

The results of the phantom study are demonstrated in Figs. 2 and 3. The average CT numbers of the contrast material in the phantom’s center were 307.0 HU in Discovery CT750 HD and 452.9 HU in Revolution CT Apex, respectively. Furthermore, as the area of the cross-section increased, the CT number was gradually decreased only in Discovery CT750 HD. However, it was preserved in Revolution CT Apex (Fig. 2). The NPS curve analysis showed that the NPS value was higher in Discovery CT750 HD than in Revolution CT Apex in low frequency range (≤ 0.15 cycle/min), and higher in Revolution CT Apex than in Discovery CT750 HD in other frequency range (Fig. 3a). MTF was higher in Revolution CT Apex than in Discovery CT750 HD in any spatial frequency; MTF_10%_ values were 0.53 cycle/min in Discovery CT750 HD and 0.63 cycle/mm in Revolution CT Apex (Fig. 3b). SDNR was higher in Revolution CT Apex than in Discovery CT750 HD in any spatial frequency (Fig. 3c).

### Participants’ demographics and radiation dose

Table 4 summarizes the participants' demographics and radiation dose. The mean participants’ height was higher in Group A than in Group B (*p* = 0.03). There was no difference in age (*p* = 0.64), sex (*p* = 0.09), body weight (*p* = 0.25), and BMI (*p* = 0.54) between the two groups. The median injected contrast volume (*p* = 0.008) and amount of iodine (*p* = 0.016) were smaller in Group B than in Group A). The median CTDI_vol_ was lower in Group B than in Group A (12.9 mGy vs. 12.4 mGy, *p* = 0.006).

**Table 4:** Participants’ demographics and radiation dose

Parameter	Group A	Group B	*p* value
Age (y)	69.9 ± 12.3	72.3 ± 13.3	0.64
Men:Women	30:8	25:16	0.09
Height (cm)	165.4 ± 7.6	161.2 ± 8.8	0.03
Body weight (kg)	63.1 ± 14.2	58.5 ± 11.8	0.25
Body mass index (kg/m^2^)	23.0 ± 4.8	22.4 ± 3.7	0.54
Contrast volume (mL)	64.0 (59.0–70.0)	56.0 (52.0–62.0)	0.008
Injected iodine (g)	15.4 (14.2–16.8)	13.7 (12.7–14.9)	0.016
CTDI_vol_ (mGy)	12.9 (12.9–12.9)	12.4 (10.0–15.6)	0.006

Data are means ± 1 standard deviation or medians with interquartile range in parentheses

*CTDI*_*vol*_ CT dose-index volume

### Quantitative image analysis

The CT numbers, background noises, and SNRs are summarized in Table 5. The median CT number of the abdominal aorta was higher in Group B than in Group A (738.9 HU vs. 818.6 HU, *p* = 0.04), while there was no difference in the thoracic aorta (762.4 HU vs. 837.5 HU, p = 0.09) and iliac arteries (674.8 HU vs. 703.6 HU, *p* = 0.19) between the two groups. There was no difference in background noises at the thoracic (22.5 HU vs. 20.5 HU, *p* = 0.11), abdominal (24.6 HU vs. 24.1 HU, *p* = 0.85), and pelvic (24.8 HU vs. 24.1 HU, *p* = 0.85) regions between the two groups. The SNR of the thoracic aorta was higher in Group B than in Group A (35.6 vs. 39.6, *p* = 0.02), while there was no difference in the SNRs of the abdominal aorta (31.5 vs. 32.7, *p* = 0.10) and iliac arteries (26.8 vs. 28.1, *p* = 0.23) between the two groups.

**Table 5:** The CT numbers, background noises, and SNRs

Parameter	Group A	Group B	*p* value
Thoracic aorta			
CT number (HU)	762.4 (702.5–854.4)	837.5 (742.5–990.6)	0.09
Background noise (HU)	22.5 (19.7–24.1)	20.5 (19.2–22.9)	0.11
SNR	35.6 (30.9–39.9)	39.6 (34.9–48.1)	0.02
Abdominal aorta			
CT number (HU)	738.9 (678.8–822.0)	818.6 (719.7–930.1)	0.04
Background noise (HU)	24.6 (22.1–27.0)	24.1 (22.1–27.1)	0.85
SNR	31.5 (26.6–34.1)	32.7 (29.5–36.8)	0.10
Iliac arteries			
CT number (HU)	674.8 (587.4–746.5)	703.6 (641.0–770.1)	0.19
Background noise (HU)	24.8 (23.7–27.9)	24.1 (22.8–27.7)	0.85
SNR	26.8 (21.4–31.0)	28.1 (23.9–31.2)	0.23

Data are medians with interquartile range in parentheses

*HU* Hounsfield Unit, *SNR* signal-to-noise ratio

### Qualitative image analysis

**Fig. 4:**
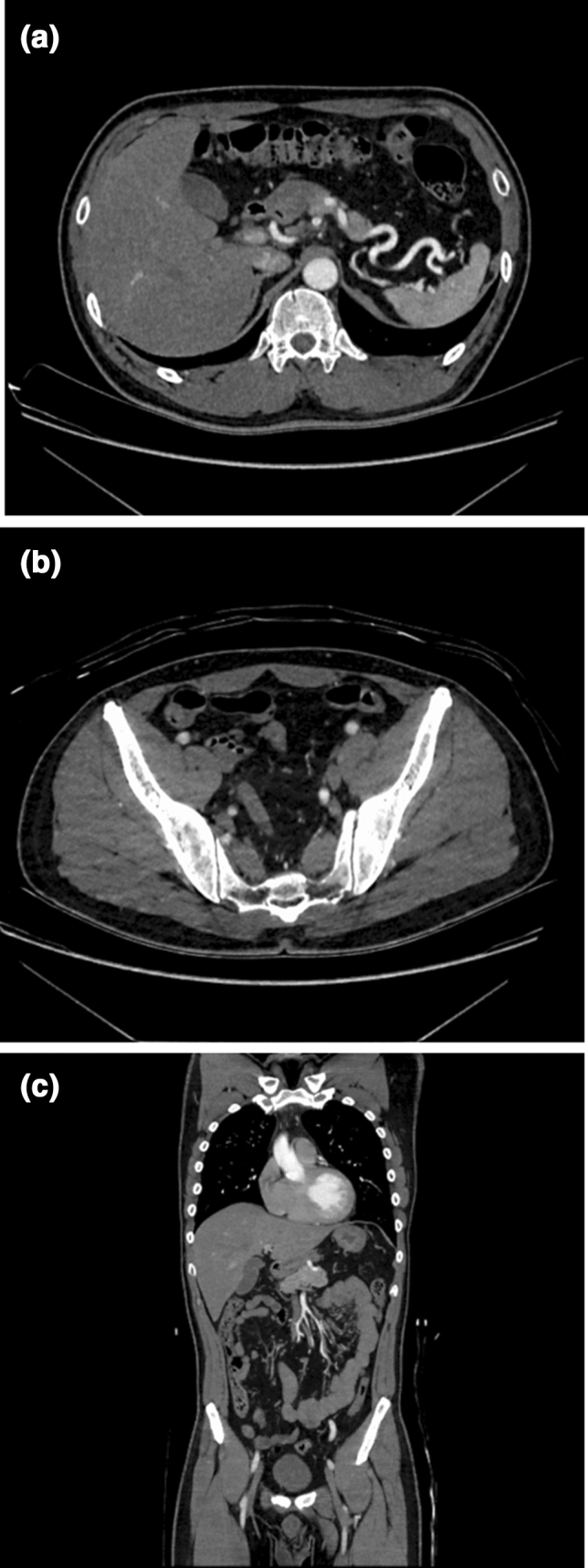
A 50-year-old man with no obvious aortic disease was scanned using Discovery CT750 HD (Group A). Axial CTA of the abdomen (**a**), pelvis (**b**), and coronal reformatted (**c**) images show the aorta and its branches clearly but also marked beam hardening artifacts

**Fig. 5:**
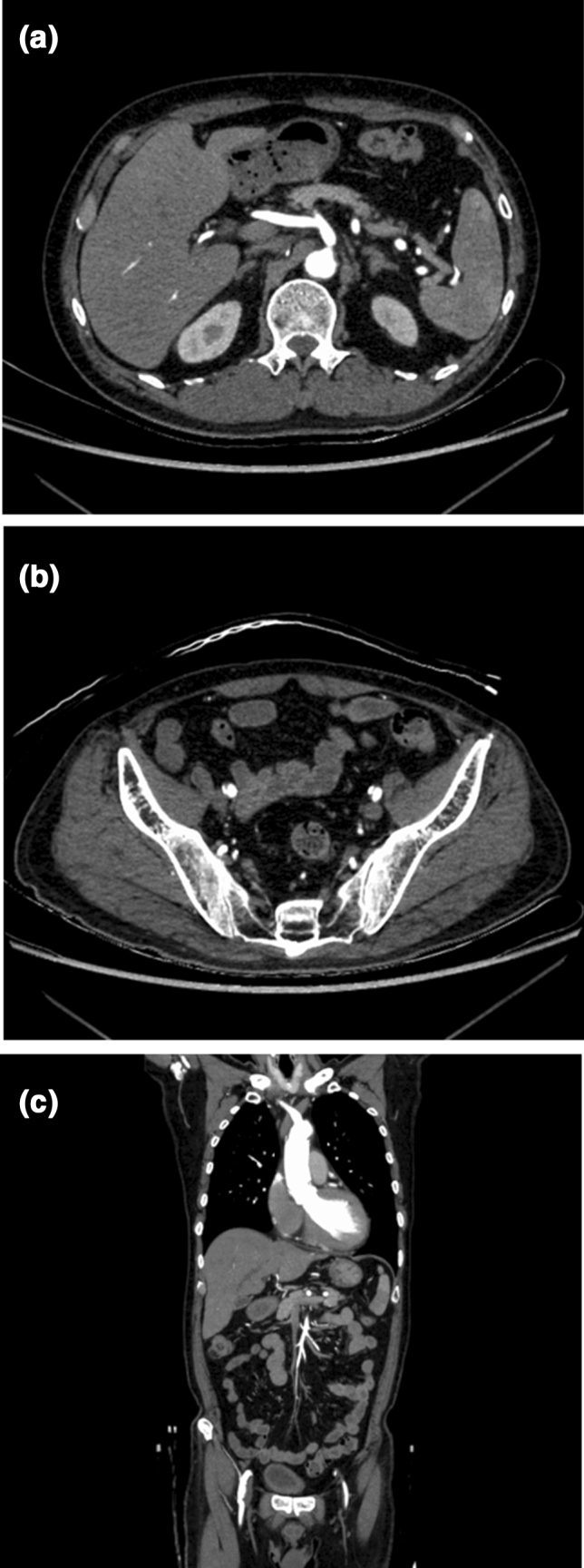
A 62-year-old man with aortic stenosis after aortic valve replacement and thoracic artificial blood vessel replacement surgery was scanned using Revolution CT Apex (Group B). Axial CTA of the abdomen (**a**), pelvis (**b**), and coronal reformatted (**c**) images clearly show the aorta and its branches. The beam hardening artifacts are improved compared to Group A (Fig. 4)

The qualitative scores for the subjective image noise, image sharpness, diagnostic acceptability, and *ĸ*-values are summarized in Table 6. Group B outperformed Group A in all parameters (*p* < 0.001–0.04) (Figs. 4 and 5). The *ĸ*-values ranged from 0.31 to 0.74, indicating fair to substantial agreement between the two radiologists.

**Table 6:** The rating scores of qualitative analyses

Parameter	Radiologist 1			Radiologist 2			*κ* value	
Image quality	Group A	Group B	*P* value	Group A	Group B	*P* value	Group A	Group B
Subjective image noise	4 (4–4)	4 (4–5)	< 0.001	4 (4–4)	5 (4–5)	< 0.001	0.53	0.65
Image sharpness	5 (4–5)	5 (5–5)	0.01	5 (4–5)	5 (5–5)	< 0.001	0.55	0.74
Diagnostic acceptability	5 (4–5)	5 (5–5)	0.04	4 (4–4)	5 (4–5)	< 0.001	0.31	0.49
**Artery depiction**								
Brachiocephalic	5 (5–5)	5 (5–5)	1.0	5 (5–5)	5 (5–5)	1.0	1.0	1.0
Common carotid	5 (5–5)	5 (5–5)	1.0	5 (5–5)	5 (5–5)	1.0	1.0	1.0
Subclavian	5 (5–5)	5 (5–5)	1.0	5 (5–5)	5 (5–5)	1.0	1.0	1.0
Bronchial	5 (4–5)	5 (4–5)	0.26	4 (4–5)	4 (3–5)	0.61	0.40	0.65
Internal thoracic	5 (5–5)	5 (5–5)	0.58	5 (5–5)	5 (5–5)	0.27	0.55	0.64
Intercostal	5 (4–5)	5 (4.75–5)	0.23	4 (4–4)	4 (4–5)	0.01	0.45	0.44
Common hepatic	5 (5–5)	5 (5–5)	0.34	5 (5–5)	5 (5–5)	0.34	1.0	1.0
Proper hepatic	5 (5–5)	5 (5–5)	0.97	5 (5–5)	5 (5–5)	0.53	0.65	1.0
Splenic	5 (5–5)	5 (5–5)	0.34	5 (5–5)	5 (5–5)	0.34	1.0	1.0
Left gastric	5 (5–5)	5 (5–5)	0.79	5 (4.5–5)	5 (4–5)	0.25	0.70	0.63
Gastroduodenal	5 (5–5)	5 (5–5)	0.34	5 (5–5)	5 (5–5)	0.44	0.65	0.88
Inferior phrenic	5 (4–5)	5 (4–5)	0.66	4 (4–5)	4 (3–5)	0.43	0.59	0.60
Superior mesenteric	5 (5–5)	5 (5–5)	0.77	5 (5–5)	5 (4.75–5)	0.52	0.55	0.50
Inferior mesenteric	5 (5–5)	5 (4–5)	0.02	5 (4–5)	4 (3.75–5)	0.047	0.37	0.58
Renal	5 (5–5)	5 (5–5)	1.0	5 (5–5)	5 (5–5)	1.0	1.0	1.0
Lumbar	5 (5–5)	5 (5–5)	0.55	5 (4–5)	5 (4–5)	0.69	0.49	0.66
Common iliac	5 (5–5)	5 (5–5)	1.0	5 (5–5)	5 (5–5)	1.0	1.0	1.0
External iliac	5 (5–5)	5 (5–5)	1.0	5 (5–5)	5 (5–5)	1.0	1.0	1.0
Internal iliac	5 (5–5)	5 (5–5)	1.0	5 (5–5)	5 (5–5)	1.0	1.0	1.0
Iliolumbar	5 (4.25–5)	5 (5–5)	0.80	4 (4–5)	5 (4–5)	0.41	0.59	0.61
Superior gluteal	5 (5–5)	5 (5–5)	0.53	5 (5–5)	5 (5–5)	0.91	0.91	0.73
Inferior gluteal	5 (4–5)	5 (5–5)	0.005	5 (4–5)	5 (5–5)	0.14	0.72	0.72
Obturator	5 (5–5)	5 (5–5)	0.66	5 (4–5)	5 (4–5)	0.58	0.65	0.52
Inferior epigastric	5 (5–5)	5 (4–5)	0.20	5 (4–5)	4 (3–5)	0.10	0.43	0.49

Data are medians with interquartile range in parentheses

The arterial depiction scores are summarized in Table 6. The depiction of the intercostal artery was better in Group B than in Group A only in radiologist 2 (*p* = 0.01), and that of the inferior gluteal artery was better in Group B than in Group A only in radiologist 1 (*p* = 0.005), and that of the inferior mesenteric artery was better in Group A than in Group B in both radiologists (*p* = 0.02 and 0.047, respectively). No difference was detected in the other arteries (*p* = 0.10–1.0). The *ĸ*-values ranged from 0.37 to 1.0, indicating fair to almost perfect agreement between the two radiologists.

## Discussion

In this study, we compared the performance of two rapid kVp-switching dual-energy CT scanners including Discovery CT750 HD and Revolution CT Apex. In phantom study, Revolution CT Apex demonstrated less beam hardening artifacts and improved spatial resolution compared with Discovery CT750 HD. Our clinical study demonstrated that CTDI_vol_ was lower in Group B (scanned by Revolution CT Apex) than in Group A (scanned by Discovery CT750 HD) under the same conditions of background noise. Injected contrast volume and amount of iodine was resultantly smaller in Group B than in Group A. The median CT numbers of the aorta and iliac arteries tended to be higher in Group B than in Group A. In terms of qualitative assessments, Group B had better image quality and nearly identical arterial depictions as Group A.

In our phantom study, average CT numbers of diluted contrast material adjusted to 150 HU at 120-kVp single-energy CT scanning with Revolution CT Apex were 307.0 HU and 452.9 HU on VMIs at 40-keV scanned by Discovery CT750 HD and Revolution CT Apex, respectively. Additionally, the CT numbers gradually decreased as the area of the cross-section increased in only Discovery CT750 HD. These findings may reflect the accuracy of beam hardening correction, which was undoubtedly influenced by faster energy separation between high- and low-energies and the 3D collimator. GE Healthcare officially announced that the Revolution CT Apex has achieved 0.25 ms cycle fast kVp-switching between 80- and 140-kVp, which is 20% higher energy separation compared to Discovery CT750 HD [14]. This technological advancement allows for less mis-registration due to motion, which leads to more efficient beam hardening correction and accurate data collection [21]. These results could be related to the results of our clinical CTA study that CT numbers of large vessels tend to be higher in Group B than in Group A. Moreover, the MTF and SDNR was higher in Revolution CT Apex than in Discovery CT750 HD in all frequency range. This means spatial resolution was higher in Revolution CT Apex and we believed this affects the results of qualitative image analyses in clinical study that Group B showed better image quality compared with Group A.

Revolution CT Apex could provide high-quality images even in conditions with comparable noise levels to Discovery CT750 HD in clinical CTA study and we believed that our results were taken by new technologies as described below. New Quantix^TM^160 X-ray tube has a powerful ability with a maximum X-ray output of 1,300 mA and 16 cm of coverage, which reduces image noise, especially in low-keV settings. Furthermore, Quantix^TM^160 supports better energy separation and synchronized kV and mA switching that is able to increase the flux associated with the low-kV projections match the high-kV projections [12, 22]. The Clarity DAS loaded in Revolution CT Apex enables a 25% reduction in electronic noise and better 80-kVp data quality by equipping with ultra-low capacitance photodiodes [14]. The 3D collimator achieved lower scattered X-rays compared to conventional 1D collimator [14]. Additionally, Revolution CT Apex achieves radiation dose reduction under comparable image noise conditions with Discovery CT750 HD, it is thought to be benefited by improved image quality and CTDI_vol_ optimization brought by the latest GSI assist.

The arterial depictions were acceptable quality in both 2 groups despite of decreased amount of iodine by about 60% on average compared to 600 mg/I, and nearly identical between the two groups, while the depiction of inferior mesenteric artery was better in Group A than in Group B. This result could be influenced by the participants’ backgrounds. That is, participants with abdominal aortic aneurysms after EVAR made up 28.8% of Group A and 46.3% of Group B. In general, EVAR is widely covering the abdominal aorta, and bifurcation of the inferior mesenteric artery would be also covered. As a result, blood flow was reduced in the proximal side of the inferior mesenteric artery, and qualitative scores for visualization of this artery were lower in Group B than in Group A.

Our study had several limitations. First, the sample size was relatively small, which could have resulted in selection bias. Second, the mean participants’ body weight and BMI in this study might be smaller than that of a western population. Third, we were unable to directly compare the two groups in a population of compatible participants and the same scan parameters. Finally, only rapid kVp-switching dual-energy CT scanners were used from a single vendor and assessed at 40-keV.

## Conclusions

Revolution CT Apex improved qualitative image quality and reduced radiation dose in dual-energy CTA at 40-keV compared with Discovery CT750 HD under the comparable image noise condition.
